# Quality of recovery after total hip and knee arthroplasty in South Africa: a national prospective observational cohort study

**DOI:** 10.1186/s12891-020-03752-x

**Published:** 2020-11-05

**Authors:** Ulla Plenge, Romy Parker, Shamiela Davids, Gareth L. Davies, Zahnne Fullerton, Lindsay Gray, Penelope Groenewald, Refqah Isaacs, Ntambue Kauta, Frederik M. Louw, Andile Mazibuko, David M. North, Marc Nortje, Glen M. Nunes, Neo Pebane, Chantal Rajah, John Roos, Paul Ryan, Winlecia V. September, Heidi Shanahan, Ruth E. Siebritz, Rian W. Smit, Simon Sombili, Alexandra Torborg, Johan F. van der Merwe, Nico van der Westhuizen, Bruce Biccard

**Affiliations:** 1 Department of Anaesthesia and Perioperative Medicine, Groote Schuur Hospital, University of Cape Town, Anzio Rd, Observatory, Cape Town, Western Cape 7925 South Africa; 2 Department of Physiotherapy, Mitchell’s Plain Hospital, AZ Berman Drive, Mitchell’s Plain, Cape Town, Western Cape 7785 South Africa; 3 Department of Anaesthesia, Paarl Hospital, Hospital Street, Paarl, Western Cape 7646 South Africa; 4 Department of Anaesthesia, Victoria Hospital, Alphen Hill Rd, Wynberg, Cape Town, Western Cape 7800 South Africa; 5 Department of Physiotherapy, New Somerset Hospital, Portswood Rd, Greenpoint, Cape Town, Western Cape 8051 South Africa; 6 Department of Physiotherapy, Universitas Academic Hospital, University of the Free State, Logeman Str, Bloemfontein, Free State 9301 South Africa; 7 Department of Physiotherapy, Victoria Hospital, Alphen Hill Rd, Wynberg, Cape Town, Western Cape 7800 South Africa; 8 Department of Orthopaedic Surgery, Mitchell’s Plain Hospital, AZ Berman Drive, Mitchell’s Plain, Cape Town, Western Cape 7785 South Africa; 9 Department of Orthopaedic Surgery, New Somerset Hospital, Portswood Rd, Greenpoint, Cape Town, Western Cape 8051 South Africa; 10 Department of Anaesthesia, Steve Biko Academic Hospital, University of Pretoria, Corner Malan and Steve Biko Str, Capital Park, Pretoria, Gauteng 0001 South Africa; 11 Department of Orthopaedic Surgery, Paarl Hospital, Hospital Street, Paarl, Western Cape 7646 South Africa; 12 Department of Orthopaedic Surgery, Groote Schuur Hospital, University of Cape Town, Anzio Rd, Observatory, Cape Town, Western Cape 7925 South Africa; 13 Department of Physiotherapy, Inkosi Albert Luthuli Central Hospital, University of KwaZulu-Natal, Vusi Mzimela Rd, Umkumbaan, Durban, KwaZulu-Natal 4091 South Africa; 14 Department of Physiotherapy, Steve Biko Academic Hospital, University of Pretoria, Corner Malan and Steve Biko Str, Capital Park, Pretoria, Gauteng 0001 South Africa; 15 Department of Anaesthesia, Grey’s Hospital, University of KwaZulu-Natal, Townbush Rd, Pietermaritzburg, KwaZulu-Natal 3201 South Africa; 16 Department of Anaesthesia, Mitchell’s Plain Hospital, AZ Berman Drive, Mitchell’s Plain, Cape Town, Western Cape 7785 South Africa; 17 Department of Orthopaedic Surgery, Inkosi Albert Luthuli Central Hospital, University of KwaZulu-Natal, Vusi Mzimela Rd, Umkumbaan, Durban, KwaZulu-Natal 4091 South Africa; 18 Department of Physiotherapy, Paarl Hospital, Hospital Street, Paarl, Western Cape 7646 South Africa; 19 Department of Physiotherapy, Grey’s Hospital, University of KwaZulu-Natal, Townbush Rd, Pietermaritzburg, KwaZulu-Natal 3201 South Africa; 20 Department of Physiotherapy, Groote Schuur Hospital, University of Cape Town, Anzio Rd, Observatory, Cape Town, Western Cape 7925 South Africa; 21 Department of Orthopaedic Surgery, Grey’s Hospital, University of KwaZulu-Natal, Townbush Rd, Pietermaritzburg, KwaZulu-Natal 3201 South Africa; 22 Department of Orthopaedic Surgery, Steve Biko Academic Hospital, University of Pretoria, Corner Malan and Steve Biko Str, Capital Park, Pretoria, Gauteng 0001 South Africa; 23 Department of Anaesthesia, Inkosi Albert Luthuli Central Hospital, University of KwaZulu-Natal, Vusi Mzimela Rd, Umkumbaan, Durban, KwaZulu-Natal 4091 South Africa; 24 Department of Orthopaedic Surgery, Universitas Academic Hospital, University of the Free State, Logeman Str, Bloemfontein, Free State 9301 South Africa; 25 Department of Anaesthesia, Universitas Academic Hospital, University of the Free State, Logeman Str, Bloemfontein, Free State 9301 South Africa

**Keywords:** Total hip arthroplasty, Total knee arthroplasty, Quality of recovery, DAH_30_, Perioperative arthroplasty practice, Enhanced recovery protocols, Low-and middle-income countries, South Africa

## Abstract

**Background:**

Encouraged by the widespread adoption of enhanced recovery protocols (ERPs) for elective total hip and knee arthroplasty (THA/TKA) in high-income countries, our nationwide multidisciplinary research group first performed a Delphi study to establish the framework for a unified ERP for THA/TKA in South Africa. The objectives of this second phase of changing practice were to document quality of patient recovery, record patient characteristics and audit standard perioperative practice.

**Methods:**

From May to December 2018, nine South African public hospitals conducted a 10-week prospective observational study of patients undergoing THA/TKA. The primary outcome was ‘days alive and at home up to 30 days after surgery’ (DAH_30_) as a patient-centred measure of quality of recovery incorporating early death, hospital length of stay (LOS), discharge destination and readmission during the first 30 days after surgery. Preoperative patient characteristics and perioperative care were documented to audit practice.

**Results:**

Twenty-one (10.1%) out of 207 enrolled patients had their surgery cancelled or postponed resulting in 186 study patients. No fatalities were recorded, median LOS was 4 (inter-quartile-range (IQR), 3–5) days and 30-day readmission rate was 3.8%, leading to a median DAH_30_ of 26 (25–27) days. Forty patients (21.5%) had pre-existing anaemia and 24 (12.9%) were morbidly obese. In the preoperative period, standard care involved assessment in an optimisation clinic, multidisciplinary education and full-body antiseptic wash for 67 (36.2%), 74 (40.0%) and 55 (30.1%) patients, respectively. On the first postoperative day, out-of-bed mobilisation was achieved by 69 (38.1%) patients while multimodal analgesic regimens (paracetamol and Non-Steroid-Anti-Inflammatory-Drugs) were administered to 29 patients (16.0%).

**Conclusion:**

Quality of recovery measured by a median DAH_30_ of 26 days justifies performance of THA/TKA in South African public hospitals. That said, perioperative practice, including optimisation of modifiable risk factors, lacked standardisation suggesting that quality of patient care and postoperative recovery may improve with implementation of ERP principles. Notwithstanding the limited resources available, we anticipate that a change of practice for THA/TKA is feasible if ‘buy-in’ from the involved multidisciplinary units is obtained in the next phase of our nationwide ERP initiative.

**Trial registration:**

The study was registered with ClinicalTrials.gov (NCT03540667).

## Background

This article reports on work from an initiative to create a unified enhanced recovery protocol (ERP) for patients undergoing elective total hip and knee arthroplasty (THA and TKA, respectively) in South Africa. The work presented follows the consensus achieved for implementation of important perioperative care principles by our nationwide multidisciplinary research group in a previously conducted Delphi study [1].

ERPs and benchmark driven registries have been introduced to most surgical specialities in high-income countries (HICs) since Professor Henrik Kehlet’s seminal work on postoperative pathophysiology and rehabilitation more than two decades ago [2]. Improved patient outcomes and better utilisation of hospital resources have resulted in care pathways for THA and TKA adopting ERPs as standard care [3–5]. However, reports on the feasibility of implementation and influence on patient outcomes in low-and middle-income countries (LMICs) are limited [6].

Firstly, initiation of benchmark-driven audits of perioperative practice and surgical outcomes for elective procedures are challenged by the ever-present burden of urgent and emergency surgeries in LMICs [7]. Secondly, constrained financial resources result in a scarcity of health professionals working in non-digitalised inefficient health care infrastructures which impedes rethinking clinical practice. South African government hospitals only have 1.1 (inter-quartal-range (IQR) 0.7–2.1) specialists per 100.000 population (unpublished data from the African Surgical Outcome Study) [8], compared with WHO’s recommendations of 20–40 specialists per 100.000 [9]. As a result, knowledge regarding quality of surgical care in LMICs, which is a prerequisite to follow in the footsteps of HICs in the implementation of ERPs, is scarce [10].

That said, we believe that it *is* possible to institute practice change in a LMIC despite limited resources and that perioperative care for THA and TKA patients in South Africa *can* improve with implementation of ERP principles. The primary objective of this second phase of changing practice was to document quality of postoperative recovery for THA and TKA patients in the South African public healthcare sector. The secondary objectives were to record patient characteristics and audit standard perioperative practice.

## Methods

The STrengthening the Reporting of OBservational studies in Epidemiology (STROBE) statement has been followed for reporting [11].

From May 8 to December 4, 2018, nine South African public hospitals conducted a 10-week prospective observational study of patients scheduled for THA or TKA. Four hospitals (150–350 beds) were District and Regional Hospitals (DRHs) while 5 were Tertiary and Central Hospitals (TCHs), characterised as highly specialised referral centres (500–850 beds). Eight of the nine hospitals participated in our previous study [1].

**Fig. 1:**
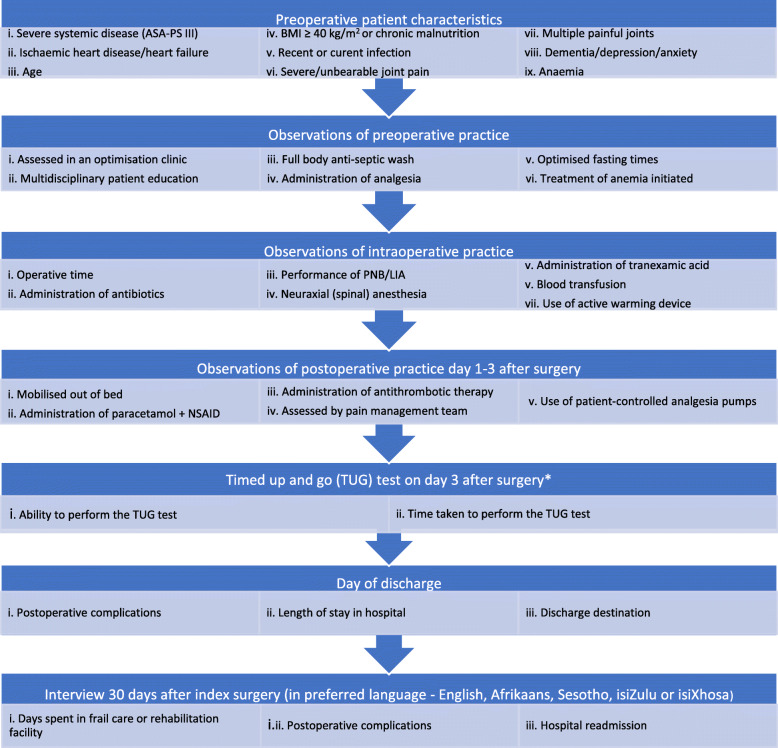
Flowchart of perioperative data capture. * TUG test was performed on day two for patients discharged before day three. If day three after surgery was on a weekend and the physiotherapist was not available to perform the test, patients would have their TUG tests on the first coming weekday i.e. day four or five after surgery

To reduce selection bias, all patients who were planned to have their THA or TKA during the specified study period at each site were assessed for eligibility to participate in the study. Patients who were older than 18 years and able to understand study questions in either English, Afrikaans, Sesotho, isiZulu or isiXhosa (as preferred) were consecutively enrolled (Fig. 1). Ability to contact the patients telephonically after hospital discharge was a prerequisite. Data were captured by staff involved in the daily care of arthroplasty patients from departments of physiotherapy, anaesthesia and orthopaedic surgery using Research Electronic Data Capture (REDCap) [12]. Thirty days after index surgery, patients were contacted telephonically to ascertain days spent in a discharge destination other than home/with family and occurrence of postoperative complications leading to hospital readmission. Local site investigators confirmed day(s) spent in hospital during readmission and assisted in retrieving missing perioperative data by manual inspection of hospital folders (definitions of postoperative complications in Supplementary data, Table S1).

### Outcome measures

In line with the objectives, the primary outcome was ‘days alive and at home up to 30 days after surgery’ (DAH_30_) [13]. DAH_30_ is a patient-centred composite endpoint incorporating early death, hospital length of stay (LOS), discharge destination and readmission during the first 30 days after surgery. As such, a lower numerical value of DAH_30_ reports fewer days spent at home/with family during the first 30 postoperative days thus quantitatively documenting impaired quality of recovery (Supplementary data, Appendix 1).

The secondary outcomes were the ability to complete and time taken to perform the ‘timed up and go’ (TUG) test on day three after surgery as measures of early functional recovery [14] (Supplementary data, Appendix 2).

Patient profile and standard perioperative practice were recorded according to the prioritised items for optimising perioperative care for THA and TKA in South Africa [1] (Supplementary data, Tables S2-S5).

As the preoperative morbidity burden and in-hospital resources vary between general and specialist hospitals, a post-hoc decision was made to examine the outcomes of the primary and secondary objectives for patients managed in DRHs compared with TCHs.

### Statistical analysis

Statistical analyses were conducted using IBM SPSS version 25 (SPSS Inc., Chicago, IL, USA).

Continuous data were presented as median (IQR), since testing for distribution of data using visual inspection of histograms, skewness, kurtosis and the Shapiro-Wilk test showed that the majority of data were not normally distributed. Comparisons were made using the Mann-Whitney U-test for non-parametric unrelated samples, and *p*-values < 0.05 were considered significant. Categorical data were expressed as frequencies and percentages, and groups were compared using Pearson’s chi-squared test or Fisher’s exact test as appropriate.

A formal sample size calculation was not performed for this descriptive hypothesis-generating study since the literature on patient characteristics, perioperative interventions and postoperative recovery after THA and TKA in South Africa and LMICs is scarce. The 10-week study period was chosen with the intention of providing a minimum of 20 patients per site, since some DRHs performed two THAs and TKAs per week at the time of protocol development.

## Results

**Fig. 2:**
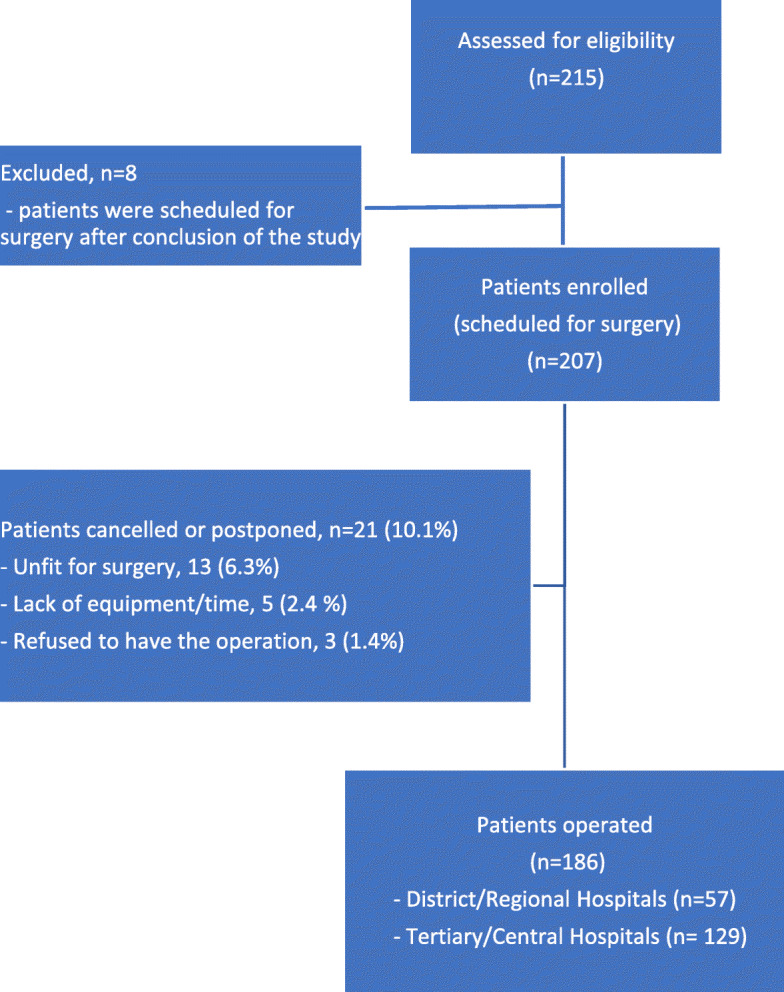
Flow diagram of the study cohort. *n* = patients

Twenty-one (10.1%) out of 207 enrolled patients had their scheduled surgery cancelled or postponed resulting in 186 study patients (Fig. 2). Patient characteristics are presented in Table 1.

**Table 1:** Baseline characteristics of total hip and knee arthroplasty patients in nine hospitals

Patient characteristics	Whole cohort (***n*** = 186)	DRHs (***n*** = 57)	TCHs (***n*** = 129)	***P***-value
**THA/TKA**	89 (47.8)/97 (52.2)	29 (50.9)/28 (49.1)	60 (46.5)/69 (53.5)	0.6
**Female**	127 (68.3)	36 (63.2)	91 (70.5)	0.3
**Severe systemic disease**^**a**^	50 (26.9)	10 (17.5)	40 (31.0)	0.06
**Ischemic heart disease**	15 (8.1)	3 (5.3)	12 (9.3)	0.6
**Heart failure**	8 (4.3)	3 (5.3)	5 (3.9)	0.7
**Age (years)**	62 (55–69)	62 (55–69)	63 (54–71)	0.6
**Chronic malnutrition**	None			
**BMI ≥ 40 kg/m**^**2**^	24 (12.9)	7 (12.3)	17 (13.2)	0.9
**Recent or current infection**	8 (4.3)	4 (7.0)	4 (3.1)	0.3
**Severe/unbearable pain**^**b**^	114 (62.0)	30 (52.6)	84 (66.1)	0.08
**Multiple painful joints**	107 (58.2)	29 (50.9)	78 (61.4)	0.2
**Dementia**	None			
**Depression/anxiety**	7 (3.8)	2 (3.5)	5 (3.9)	1.0
**Anaemia**^**c**^	40 (21.5)	9 (15.8)	31 (24.0)	0.2

Data are n (%) or median (IQR)

^a^American Society of Anesthesiologists Physical Status (ASA-PS) III

^b^Functional pain in joint to be operated

^c^Female Hgb < 12 g/dl and male Hgb < 13 g/dl

Please see the Abbreviations section for explanation of the acronyms and Supplementary data, Table S2 for definitions of patient characteristics

**Fig. 3:**
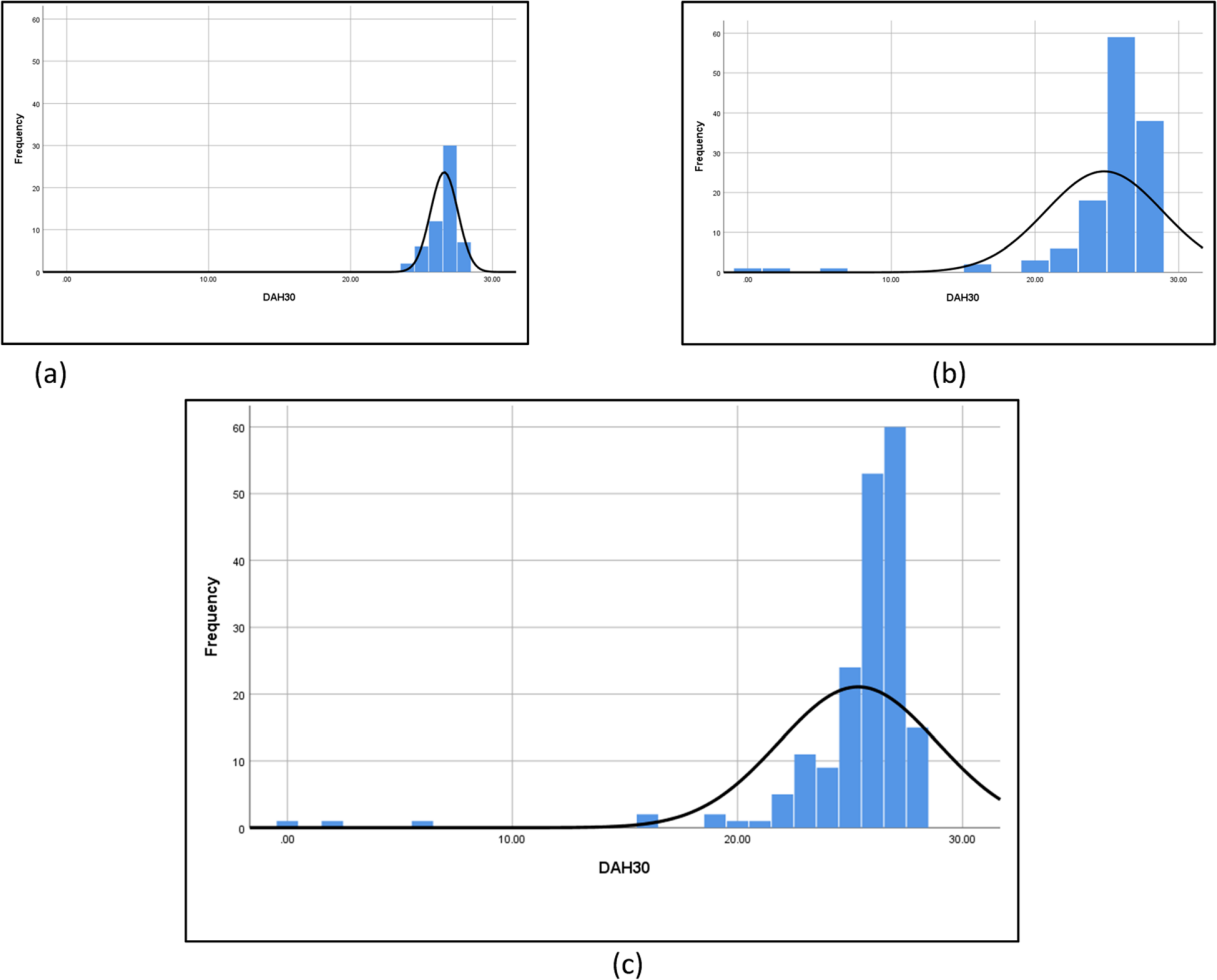
Distribution of DAH_30_. **a** DAH_30_ for District and Regional Hospitals (*n* = 57). **b** DAH_30_ for Tertiary and Central Hospitals (*n* = 129). **c** DAH_30_ for the full patient cohort (*n* = 186). Y-axis = Number of patients, X-axis = Days alive and at home up to 30 days after surgery (DAH_30_). The smoothing line represents normal distribution

**DAH**_**30**_**.** Data capture was complete for all components of DAH_30_. Median (IQR) LOS in hospital was 4 (3–5) days with significantly shorter stay in DRHs [3 (3, 4) vs 4 (3–5) days, *p* <  0.001]. One hundred and seventy-one patients (98.8%) mobilised independently with assistive devices on day of discharge and only six patients (3.2%) were transferred to frail care or rehabilitation. No fatalities were encountered but one patient remained hospitalised during the 30-day study period after falling and requiring joint revision of her newly operated hip. Twelve patients (6.5%) developed postoperative complications [1 (1.8%) in DRHs vs 11 (8.5%) in TCHs, *p* = 0.1], (Table 2), and seven patients (3.8%) were readmitted to hospital [none from DRHs vs 7 (5.4%) from TCHs, *p* = 0.1]. The distribution of DAH_30_ was left-skewed, with a median of 26 (25–27) days (Fig. 3.c). Patients receiving surgery in DRHs had a median DAH_30_ of 27 (26, 27) days compared with 26 (24–27) days in TCHs, *p* <  0.001 (Fig. 3.a and 3.b, respectively).

**Table 2:** Thirty-day complication rate after total hip and knee arthroplasty in nine hospitals

30-day postoperative complications	Whole cohort (***n*** = 186)	DRHs (***n*** = 57)	TCHs (***n*** = 129)	***P***-value
**Patients with postoperative complications**	12 (6.5)	1 (1.8)	11 (8.5)	0.1
**● Minor procedural complications**^**a**^	5 (2.7)	1 (1.8)	4 (3.1)	1.0
**● Pulmonary Emboli**	1 (0.5)	None	1 (0.8)	1.0
**● Troponin T leak**	1 (0.5)	None	1 (0.8)	1.0
**● Postoperative blood transfusion**	1 (0.5)	None	1 (0.8)	1.0
**● Joint revision**	5 (2.7)	None	5 (3.9)	0.3
**●Joint dislocation**^**b**^	3 (1.6)	None	3 (2.3)	0.6
**●Periprosthetic joint infection**^**c**^	2 (1.1)	None	2 (1.6)	1.0

Data are n (%)

1 patient had 2 postoperative complications (in-hospital wound oozing and readmission with pulmonary emboli)

^a^For example wound oozing, wound haematoma

^b^Only total hip arthroplasties

^c^Only total knee arthroplasties

Please see the Abbreviations section for explanation of the acronyms and Supplementary data, Table S1 for definitions of postoperative complications

### Functional recovery

Data were missing for eight patients (4.3%). All 22 patients (12.4%) who were discharged before day three completed their TUG test, whereas 17/118 patients (14.4%) and 1/38 patients (2.6%) were unable to perform the test on day three and day four/five, respectively. The time taken to perform the test on day three was significantly shorter in DRHs (*p* = 0.02), (Supplementary data, Table S6).

### Perioperative practice

Perioperative observations are shown in Table 3 and Supplementary data, Table S7.

**Table 3:** Perioperative practice for total hip and knee arthroplasty patients in nine hospitals

Perioperative practice	Whole cohort (***n*** = 186)	DRHs (***n*** = 57)	TCHs (***n*** = 129)	***p***-value
**Preoperative**				
**Patient attended an optimisation clinic**	67 (36.2)	13 (22.8)	54 (42.2)	0.01
**Patient received multidisciplinary education**	74 (40.0)	25 (43.9)	49 (38.3)	0.5
**Patient had full body anti-septic wash**	55 (30.1)	12 (21.1)	43 (34.1)	0.07
**Patient received analgesia**	125 (67.9)	31 (54.4)	94 (74.0)	0.01
**Clear fluids provided 2–6 h before surgery**	37 (20.0)	8 (14.0)	29 (22.7)	0.2
**Solids provided 6–10 h before surgery**	42 (23.1)	18 (31.6)	24 (19.2)	0.07
**Treatment of anaemia initiated**^**a**^	4/40 (10)	None	4 (12.9)	0.6
**Intraoperative**				
**THA: Operative time (minutes)**	90 (75–106)	91 (80–118)	90 (71–106)	0.6
**TKA: Operative time (minutes)**	105 (85–125)	113 (97–126)	100 (73–125)	0.06
**Antibiotics ≤ 30 min before skin cut**	185 (100)	57 (100)	128 (100)	
**THA: Performance of PNB and/or LIA**	30 (36.1)	15 (60.0)	15 (25.9)	0.003
**TKA: Performance of PNB and/or LIA**	62 (66.7)	26 (92.9)	36 (55.4)	< 0.001
**Neuraxial (spinal) anaesthesia**	121 (65.1)	47 (82.5)	74 (57.4)	0.001
**Administration of tranexamic acid**	178 (96.2)	57 (100)	121 (94.5)	0.1
**Blood transfusion**	1 (0.5)	None	1 (0.8)	1.0
**Use of active warming device**	179 (97.3)	55 (96.5)	124 (97.6)	0.6
**Postoperative day 1**				
**Patient mobilised out of bed**	69 (38.1)	36 (65.5)	33 (26.2)	< 0.001
**Patient received paracetamol + NSAID**^**b**^	29 (16.0)	14 (25.5)	15 (11.9)	0.02
**Patient received antithrombotic therapy**^**b**^	149 (85.1)	46 (85.2)	103 (85.1)	1.0
**Patient assessed by pain management team**	103 (57.5)	32 (59.3)	71 (56.8)	0.8
**Use of patient-controlled analgesia**^**b**^	82 (45.3)	23 (41.8)	59 (46.8)	0.5

Data are *n* (%) or median (IQR)

*a* = 40 patients (21.5%) presented with anaemia, 4 (10.0%) had initiated medical treatment to increase haemoglobin concentration; *b* = drugs administered since conclusion of surgery

Please see the Abbreviations section for explanation of the acronyms and Supplementary data, Tables S2-S5 for definitions of perioperative interventions and Table S8 for reporting of missing data

The preoperative period was characterised by limited implementation of measures to prepare patients for surgery. Sixty-seven patients (36.2%) were assessed in an optimisation clinic (*p* = 0.01 in favour of TCHs), multidisciplinary education was offered to 74 patients (40.0%) while full body anti-septic wash and optimised fasting regimens were implemented for less than a third of all patients.

Recordings of median operative times were 90 (75–106) minutes for THAs and 105 (85–125) minutes for TKAs with no significant difference between DRHs and TCHs for either procedure. Three intraoperative care principles were implemented for > 95% of the study population; antimicrobial prophylaxis (100%), prevention of perioperative blood loss with anti-fibrinolytics (96.2%) and maintaining normothermia (97.3%). Conversely a peripheral nerve block and/or local infiltration analgesia (PNB/LIA) were performed for 30 THA patients (36.1%) and 62 TKA patients (66.7%). Intergroup analysis showed significantly more TKA patients in DRHs received PNB and/or LIA (26, 92.9%; *p* < 0.001).

On the first postoperative day, out-of-bed mobilisation was achieved by 69 patients (38.1%) while multimodal opioid-sparing analgesic regimens with paracetamol and Non-Steroid-Anti-Inflammatory-Drugs (NSAIDs) were implemented for 29 patients (16.0%) (both significantly more frequent in DRHs). Although pain management teams assessed 103 patients (57.5%) on the first postoperative day, only 11 patients (7.0%) continued treatment with paracetamol and NSAIDs till the third postoperative day. Postoperative thromboprophylaxis was implemented for 80–85% of all patients on the first 3 days after surgery.

Documentation of missing data in Supplementary data, Table S8.

## Discussion

This observational multicentre study has informed us that 30-day quality of recovery measured by DAH_30_ of 26 days justifies performance of THA and TKA in the South African public healthcare sector. However, the cancellation/postponement rate was high, postoperative functional recovery was delayed and perioperative practice including optimisation of modifiable risk factors lacked standardisation. The findings from this second study towards changing practice for THA and TKA patients in South Africa suggest that a nationwide initiative to implement ERP principles may improve quality of patient care and postoperative recovery.

### The quality of postoperative recovery for THA and TKA patients

Both in-hospital and post-discharge qualitative and quantitative measures should ideally be reported to describe a patient’s recovery trajectory fully following surgery [15]. Our primary outcome, DAH_30_, provided such information for the first 30 days after surgery by merging patient-centred quality of recovery with objectively recorded quantitative outcomes [13].

DAH_30_ from our study of 26 days was similar to ‘days alive and out of hospital at 30 days after surgery’ in a Canadian study with > 280.000 THA and TKA patients [16] but inferior to a Danish study with > 16.000 patients operated within a fast-track protocol [17]. Although ‘days out of hospital’ does not account for time spent in a postoperative frail care or rehabilitation facility, neither of the two settings routinely use step down facilities [17, 18] which is why their results can be compared with our DAH_30_. The observation that a higher DAH_30_ was accomplished in fast-track settings is supported by results from a population-based study including > 1.5 million THA and TKA patients [19], where a greater utilisation of enhanced recovery components was associated with fewer complications and shorter LOS in hospital. When comparing results according to hospital level, a higher burden of comorbidities [16, 17, 20] and delayed mobilisation [19, 21] may have contributed to the significantly lower DAH_30_ observed for patients operated in TCHs.

Hospital readmissions (3.8%) and 30-day postoperative complications (6.5%) reflects quality of early recovery and result in fewer days spent at home/with family. Both variables are contained in DAH_30_ and both were similar to international data [18, 22–24]. However, our joint revisions accounted for 2.7% of the complications (three joint dislocations following THA (3.4%) and two periprosthetic infections following TKA (2.1%)) which is greater than 30-day THA and TKA data from HICs [17, 22].

Achieving early mobilisation after THA and TKA reflects functional recovery and return of homeostasis. Early ambulation is thus associated with reduced LOS [21] and is, as part of an ERP, believed to reduce postoperative complications by hindering the adverse physiological effects of bed rest [2, 19, 25]. Contrary to ERP goals of same day (as operation) mobilisation [26], only 38.1% of our study cohort mobilised out of bed on the first postoperative day while 14.4% of patients were unable to complete the TUG test on the third postoperative day. Inability to perform timely joint replacement is associated with musculoskeletal decompensation and delayed rehabilitation in LMICs [27]. Lack of standardised postoperative multimodal pain management, as was the case in our study, possibly further contributed to delayed mobilisation [26]. It was however noteworthy that patients operated in DRHs who were more likely to mobilise out of bed on the first postoperative day (*p* < 0.001), performed the TUG test faster on the third postoperative day (*p* = 0.02) making a case for enhanced mobilisation programmes in a setting like ours.

### The patient characteristics and standard perioperative practice

ERPs were originally introduced as procedure specific evidence-based recommendations aiming to reduce the surgical stress response and enhance postoperative recovery [2]. However, in today’s arthroplasty practice, ERP components have been adopted from other surgical specialities (i.e. not procedure specific recommendations) and discrimination between standard care for modern surgical practice and ERP principles is less pronounced [28]. It follows, that the enhanced care programme developed by our multidisciplinary group which determined the design of this study also contained components considered standard practice in HICs [1].

Only three intraoperative care principles identified as being important for improving postoperative outcomes in our previous study were consistently implemented for more than 95% of patients; i. antimicrobial prophylaxis, ii. tranexamic acid to reduce perioperative blood loss and iii. Measures to maintain normothermia. Postoperative thromboprophylaxis treatment was instituted for 80–85% of our study population the first 3 days after surgery while all other components pertaining to patients’ perioperative journey were delivered with great variation.

However, informed of this inconsistency in patient care, we are now equipped to change practice and facilitate implementation of preoperative multidisciplinary involvement with the aim to ensure patients are physiologically and mentally fit for surgery (i.e. utilise the preoperative period as a ‘window of opportunity’) [29]. Such improvements should enhance patient safety (21.5% of our patients were anaemic [30] and 12.9% were morbidly obese with Body Mass Index (BMI) ≥ 40) [31]) and resource utilisation (10.1% of patients were cancelled/postponed prior to surgery). Further, in our setting with low literacy and limited ability to seek information, we are hopeful that a pragmatic approach to patient education would improve patient empowerment and participation in postoperative rehabilitation, as is standard practice in HICs [28]. Such measures along with implementation of anti-septic body wash and optimised fasting regimens would likely prepare patients better for their scheduled arthroplasty.

We now also have a strong case to educate patients, nurses and doctors in the importance of perioperative multimodal opioid-sparing analgesic regimens to facilitate early postoperative mobilisation. Both are considered core elements in ERPs for joint replacements [32] but with the exception of PNB/LIA for TKA in DRHs, neither were consistently implemented in our practice.

Finally, length of surgery is associated with postoperative complications and is as such a measure of quality of surgery [33, 34]. In support of the quality of surgery provided in our public hospitals, median operative times of 90 and 105 min for THA and TKA, respectively, were similar to results from US and Canadian databases [33, 34]. Although wide IQRs informed of great variation in the performance of joint replacements in both DRHs and TCHs, we believe that such variation in operative times was likely to be associated with the degree of pathology encountered, which in our setting with year-long waiting lists, is often severe. Consequently, we are confident that the quality of surgery was comparable to that of HICs.

Our study had many strengths. Firstly, eight out of nine involved sites had participated in our previous study which possibly heightened accountability for quality of data capture and limited missing data. We thus succeeded in creating a strong tracking system, with 100% data capture for our primary outcome. Secondly, we documented patients’ ability to mobilise independently before discharge. This allowed us to ensure that patients were not inappropriately discharged before adequate functional recovery which would have falsely improved the DAH_30_. We are therefore confident in the primary outcome reported. Thirdly, in support of the documented observations, 12 out of 16 strong recommendations for implementation from Enhanced Recovery After Surgery (ERAS) Society’s newly published consensus statement for THAs and TKAs were investigated [35]. Use of patient-centred outcome measures like DAH_30_ are furthermore recommended [36]. Finally, presenting the data according to hospital level has given us an appreciation of resources and challenges pertaining to hospital category. This will assist in facilitating change across different hospital levels.

Our study also had limitations. An observational study inherently carries a risk of selection bias, however, all patients scheduled for THA and TKA during the dedicated study period were invited to participate and with minimal exclusion criteria, we believe selection bias was low. Cognisant of the risk of ‘investigator fatigue’ in a non-funded study like ours, we designed a 10-week study to enrol a minimum of 20 patients per site. However, affected by the high cancellation/postponement rate and unexpected reduction in performance of elective joint replacements at certain sites (as a result of burden of non-elective surgery and/or shortage of personnel), only three hospitals (one DRH and two TCHs) succeeded in operating the minimum of 20 patients. While this observation illustrates ‘real-time’ challenges of providing timely joint arthroplasties in our setting, limited patient numbers can compromise external validity of the study results. However, by involving different level hospitals from four South African provinces, we believe our audit of perioperative practice and postoperative quality of recovery is representative for patients undergoing THA or TKA in the South African public healthcare sector.

## Conclusion

Quality of recovery measured by a median DAH_30_ of 26 days justifies performance of THA and TKA in the South African public healthcare sector. That said, functional recovery was delayed and perioperative practice including optimisation of pre-existing modifiable risk factors lacked standardisation. These findings suggest that quality of patient care and postoperative recovery may improve with implementation of ERP principles. Notwithstanding the limited resources available in a LMIC like South Africa, we anticipate that a change of practice for THA and TKA is feasible if ‘buy-in’ from the involved multidisciplinary units is obtained in the next phase of our nationwide ERP initiative.

## Supplementary Information

**Additional file 1.**

## Data Availability

The datasets used and analysed during the current study are available from the corresponding author on reasonable request.

## Abbreviations

ERP: Enhanced recovery protocols
HICs: High-income countries
THA: Total hip arthroplasty
TKA: Total knee arthroplasty
LMICs: Low-and middle-income countries
STROBE: The STrengthening the Reporting of OBservational studies in Epidemiology
DRHs: District and regional hospitals
TCHs: Tertiary and central hospitals
REDCap: Research Electronic Data Capture
DAH_30_: Days alive and at home up to 30 days after surgery
LOS: Length of stay
TUG: Timed up and go
PNB: Peripheral nerve block
LIA: Local infiltration analgesia
NSAID: Non-Steroid-Anti-Inflammatory-Drugs
BMI: Body Mass Index
ERAS: Enhanced Recovery After Surgery.
