# Supplementary material for: Quality of recovery after total hip and knee arthroplasty in South Africa: a national prospective observational cohort study
Source: BMC Musculoskelet Disord. 2020 Nov 5;21:721. doi: 10.1186/s12891-020-03752-x (PMC7643442; doi:10.1186/s12891-020-03752-x)
Supplement: Supplementary file 1 — Additional file 1. [file 12891_2020_3752_MOESM1_ESM.docx]

**Supplementary data**

**Appendix A**

**Method for calculating ‘days alive and at home up to 30 days after surgery’ (DAH_30_)**

DAH_30_ is a composite endpoint incorporating early death, days spent in hospital, days spent in frail care/rehabilitation after discharge from hospital and readmission during the first 30 days after surgery.

In accordance with the paper by Myles and colleagues [1], day of surgery was assigned as day 0. If patients were readmitted, LOS during readmission was combined with LOS after index surgery. Days spent in frail care or rehabilitation facility were added to the total hospital stay. For example, if patients were discharged on day 3 after surgery and readmitted for 4 days before their second discharge, they were assigned 23 DAH_30_. If patients never left hospital or were discharged to nursing home/rehabilitation after index surgery before day 30, then patients would be assigned 0 DAH_30._ If patients died at any time during the 30-day period, they would be assigned 0 DAH_30._

**Appendix B**

**Method for calculating ‘timed up and go’ (TUG) test**

On day 3 after surgery, the physiotherapist performed a TUG test [2]. A TUG test assesses the ability to complete and time taken to raise independently from a chair, walk 3 meters with walking device and return to sitting position in the same chair. Patients discharged earlier than day 3 had the TUG test on day 2 (no patients were discharged on day 1). Patients who were not able to have their TUG test during the weekend due to lack of physiotherapy staff, had their test done first coming Monday (day 4 or 5 for patients operated on Thursdays and Fridays, respectively). Patients who were unable to perform the TUG test as scheduled were registered as unsuccessful. The TUG test was recorded in seconds.

**Table S1**

Definitions of medical and surgical complications recorded during index admission and/or readmission within 30 days from index surgery [3].

| Myocardial infarction | Increase in serum cardiac biomarker values (preferably cardiac troponin) with at least one value above the 99th percentile upper reference limit **and** at least one of the following criteria:   - Symptoms of ischaemia - New or presumed new ST-segment or T-wave ECG changes or new left bundle branch block - Development of pathological Q-waves on ECG - Radiological or echocardiographic evidence of new loss of viable myocardium or new regional wall motion abnormality - Identification of an intra-coronary thrombus at angiography or autopsy |
| --- | --- |
| Troponin leak | Absolute level of Troponin T ≥ 0.03 ng/mL |
| Deep vein thrombosis | Formation of a blood clot or thrombus within the venous system verified with ultrasound and/or CT-scan |
| Pulmonary Emboli (PE) | A new blood clot or thrombus within the pulmonary arterial system. Appropriate diagnostic tests include scintigraphy and CT angiography. Plasma D-dimer measurement is not recommended as a diagnostic test in the first three weeks following surgery. |
| Urinary Tract Infection | An infection associated with at least one of the following signs or symptoms which should be identified within a 24-hour period;   - Fever (>38 °C), urgency, frequency, dysuria, suprapubic tenderness, costovertebral angle pain or tenderness with no other recognised cause - AND a positive urine culture of ≥105 colony forming units/mL with no more than two species of microorganisms |
| Pneumonia | Chest radiographs with new or progressive and persistent infiltrates, or consolidation, or cavitation, and at least one of the following:   - Fever (> 38°C) with no other recognized cause - Leukopenia (<4,000 white blood cells/mm^3^) or leucocytosis (>12,000 white blood cells/mm^3^) - For adults >70 years old, altered mental status with no other recognised cause;   and at least two of the following:   - New onset of purulent sputum or change in character of sputum, or increased respiratory secretions, or increased suctioning requirements - New onset or worsening cough, or dyspnoea, or tachypnoea - Rales or bronchial breath sounds - Worsening gas exchange (hypoxaemia, increased oxygen requirement or increased ventilator demand)   Guidance: Two radiographs are required for patients with underlying pulmonary or cardiac disease. The definition may be used to identify ventilator associated pneumonia. |
| Blood transfusion | Administration of packed red blood cells. |
| Minor procedural complications | For example, wound oozing, wound hematoma. |
| Surgical complications requiring joint revision | For example, joint dislocation, periprosthetic infection or loosening of prosthesis leading to joint revision. |
| Surgical Site Infection (Superficial) | Infection involving only superficial surgical incision which meets the following criteria:  1. Infection occurs within 30 days after surgery and  2. Involves only skin and subcutaneous tissues of the incision and  3. The patient has at least one of the following:   - purulent drainage from the superficial incision - organisms isolated from an aseptically obtained culture of fluid or tissue from the superficial incision and at least one of the following signs or symptoms of infection: pain or tenderness, localized swelling, redness, or heat, or superficial incision is de deliberately opened by surgeon and is culture positive or not cultured. A culture-negative finding does not meet this criterion - diagnosis of an incisional surgical site infection by a surgeon or attending physician |
| Surgical Site Infection (Deep) | An infection which involves both superficial and deep parts of surgical incision and  meets the following criteria:   - Infection occurs within 30 days after surgery if no surgical implant is left in place or one year if an implant is in place and - The infection appears to be related to the surgical procedure and involves deep   soft tissues of the incision (e.g. fascial and muscle layers) and   - The patient has at least one of the following:      1. purulent drainage from the deep incision but not from the organ/space component of the surgical site 2. a deep incision spontaneously dehisces or is deliberately opened by a surgeon and is culture-positive or no cultures were taken whilst the patient has at least one of the following signs or symptoms of infection: fever (>38°C) or localized pain or tenderness. A culture-negative finding does not meet this criterion 3. an abscess or other evidence of infection involving the deep incision is found on direct examination, during surgery, or by histopathologic or radiologic examination 4. diagnosis of a deep incisional surgical site infection by a surgeon or attending physician |
| Acute Kidney Injury | - Increase of serum creatinine to 3 times baseline value OR - Increase of serum creatinine to ≥ 354 µmol/L OR - Initiation of renal replacement |
| Stroke | Embolic, thrombotic or haemorrhagic cerebral event with transient OR persistent residual motor, sensory or cognitive dysfunction (e.g. hemiplegia, hemiparesis, aphasia, sensory deficit, impaired memory). Verified by neurologist assessment and/or CT or MRI. |

**Table S2**

Definition of prioritised *preoperative risk factors* considered most important determinants of poor outcomes in patients scheduled for primary elective unilateral total hip and knee arthroplasty in South Africa [4].

| Variable | Definition |
| --- | --- |
| 1. Poor general health | Severe systemic disease classified as ASA-PS III. |
| 2. Impaired cardiovascular functional status | History of Ischaemic heart disease defined as; i) stable/unstable angina, ii) previous myocardial infarct. And/or history of heart failure defined as; patients with clinical signs of cardiac failure or patients on anti-congestive treatment. |
| 3. Advanced age | Age in years. |
| 4. Preoperative mobility | Not assessed in this study. |
| 5. Obesity or chronic malnutrition | Obesity; BMI ≥ 40 kg/m^2^. Chronic malnutrition; i. BMI < 16 kg/m^2^ for more than 3 months, and/or ii. serum albumin <30 g/L, and/or iii. emaciation and/or iv. muscle wasting/adipose wasting. |
| 6. Recent or current infection | ≤ 2 weeks of infection before surgery from UTI, RTI, dental, skin or GI based on clinical examination and objective markers. |
| 7. Preoperative chronic pain | Severe/unbearable functional pain in joint to be operated during activity. And/or multiple painful joints. |
| 7. Matching surgical complexity with surgical experience or skill | Not assessed in this study |
| 9. Psychiatric disorders and or cognitive impairment | Diagnosed with depression and/or anxiety according to ICD-10. Dementia diagnosed as cognitive impairment, most commonly patient will be in the care of geriatric team. |
| 10. Preoperative anemia | Hgb < 12 g/dL for female and Hgb < 13 g/dL for male |

* ASA-PS = American Society of Anesthesiologists Physical Status

**Table S3**

Definitions of prioritised *preoperative interventions* considered most important determinants to improve outcomes following primary elective unilateral total hip and knee arthroplasty in South Africa [4].

| Variable | Definition |
| --- | --- |
| 1. A patient optimisation clinic | Patient assessed in a formal preoperative anaesthetic clinic or by a medical/anaesthetic consultant earlier than 1 day before surgery. |
| 2. Multidisciplinary planning | Not assessed in this study |
| 3. Patient education | Formal multidisciplinary education provided (any format - written/electronic/education clinic by any combination of nursing staff/physiotherapist/social worker/occupational therapist/  anaesthetist/orthopaedic surgeon). |
| 4. Infection prevention | Full body anti-septic wash the night before or morning of surgery. |
| 5. Establishing high-volume units | Not assessed in this study. |
| 6. Smoking cessation | Not assessed in this study. |
| 7. Optimization of preoperative analgesia regimen | Administration of any analgesia the night before or morning of surgery. |
| 8. Optimize preoperative fasting times | i) Oral intake 2-6 hours before surgery and ii) solids 6-10 hours before surgery. |
| 9. Establish a patient blood management programme | If anaemic, was medical treatment initiated prior to surgery. |
| 10. Alcohol cessation | Not assessed in this study. |

**Table S4**

Definitions of prioritised *intraoperative interventions* considered most important determinants to improve outcomes following primary elective unilateral total hip and knee arthroplasty in South Africa [4].

| Variable | Definition |
| --- | --- |
| 1. Meticulous surgical technique | Operative time defined by time from knife on skin till dressing on. |
| 2. Infection prevention | Antibiotics administered within 30 min from start of surgery. |
| 3. Optimisation of prosthesis choice and placement | Not assessed in this study. |
| 4. Multimodal opioid-sparing analgesia regimen | i) Peripheral nerve block performed by anaesthetist and/or local infiltration analgesia performed by surgeon. |
| 5. Monitoring and optimisation of haemodynamics | Not assessed in this study. |
| 6. Central neuraxial anaesthesia | Spinal anaesthesia with/without opioids. |
| 7. Establish a patient blood management programme | i) Administration of tranexamic acid, ii) blood transfusion. |
| 8. Temperature regulation | Use of active warming device. |
| 9. Glycaemic control | Not assessed in this study. |
| 10. Deep vein thrombosis prophylaxis | Not assessed in this study. |

**Table S5**

Definitions of prioritised *postoperative interventions* considered most important determinants to improve outcomes following primary elective unilateral total hip and knee arthroplasty in South Africa [4].

| Variable | Definition |
| --- | --- |
| 1. Early mobilisation after surgery | Out of bed mobilisation with/without assistive device. |
| 2. Standardised orthopaedic nursing care | Not assessed in this study. |
| 3. Multimodal opioid-sparing analgesia regimen | Administration of paracetamol combined with Non-Steroid-Anti-Inflammatory-Drugs. |
| 4. Active management of medical co-morbidities | Not assessed in this study. |
| 5. DVT prophylaxis | Administration of acetylsalicylic acid or enoxaparin. |
| 6. A pain management team | Assessed by pain team. |
| 7. Patient empowerment in his or her recovery | Not assessed in this study. |
| 8. Patient controlled analgesia | Use of patient-controlled analgesia (PCA). |
| 9. Multidisciplinary ward rounds | Not assessed in this study. |
| 10. Postoperative rehabilitation | Not assessed in this study. |

**Table S6**

Postoperative ‘timed up and go’ (TUG) test for total hip and knee arthroplasty patients in nine hospitals, comparing District/Regional Hospitals with Tertiary/Central Hospitals.

| **TUG test** | **Whole cohort (n=186)** | **DRHs (n=57)** | **TCHs (n=129)** | **P-value** |
| --- | --- | --- | --- | --- |
| **Day 2^a^** | 59s (37-85) | 44s (37-112) | 63s (37-85) | 1.0 |
| **Day 3^b^** | 66s (44-99) | 47s (34-76) | 71s (46-108) | 0.02 |
| **Day 4/5^c^** | 74s (38-131) | 59s (38-83) | 82s (38-155) | 0.3 |

Median (IQR); s= seconds.

8 patients (4.3%) did not perform a TUG assessment.

a = 22 patients performed the TUG test; b = 101/118 patients performed the TUG test; c =37/38 patients performed the TUG test.

**Table S7**

Perioperative practice on the second and third postoperative day for total hip and knee arthroplasty patients in nine hospitals, comparing District/Regional Hospitals with Tertiary/Central Hospitals [4].

| **Observations of interventions** | **Whole cohort (n=186)** | **DRHs**  **(n=57)** | **TCHs**  **(n=129)** | **P-value** |
| --- | --- | --- | --- | --- |
| **Day 2** | | | | |
| **Patient mobilised out of bed** | 149 (85.1) | 48 (88.9) | 101 (83.5) | 0.4 |
| **Patient received paracetamol + NSAID ≤ 24 h** | 25 (14.3) | 11 (20.4) | 14 (11.6) | 0.1 |
| **Patient received antithrombotic therapy ≤ 24 h** | 149 (85.1) | 46 (85.2) | 103 (85.1) | 1.0 |
| **Patient assessed by pain management team** | 60 (35.1) | 32 (61.5) | 28 (23.5) | <0.001 |
| **Use of patient-controlled-analgesia ≤ 24 h** | 20 (11.7) | 9 (17.3) | 11 (9.2) | 0.1 |
| **Day 3** | | | | |
| **Patient mobilised out of bed** | 149 (94.9) | 38 (90.5) | 111 (96.5) | 0.2 |
| **Patient received Paracetamol + NSAID ≤ 24 h** | 11 (7.0) | 3 (7.0) | 8 (7.0) | 1.0 |
| **Patient received antithrombotic therapy ≤ 24 h** | 128 (81.5) | 36 (85.7) | 92 (80.0) | 0.4 |
| **Patient assessed by pain management team** | 60 (35.1) | 32 (61.5) | 28 (23.5) | <0.001 |
| **Use of patient-controlled-analgesia ≤ 24 h** | 20 (11.7) | 9 (17.3) | 11 (9.2) | 0.1 |

Data are n (%) or median (IQR). Please see the Abbreviations section for definitions of acronyms.

**Table S8**

Missing data for patient characteristics and perioperative data (n = 186).

| **Patient profile** | **Missing patient data** | | |
| --- | --- | --- | --- |
| **Poor general health** | **None** | | |
| **Impaired cardiovascular functional status** | **None** | | |
| **Advanced age** | **None** | | |
| **Obesity or chronic malnutrition** | **None** | | |
| **Recent or current infection** | **None** | | |
| **Preoperative chronic pain** | **2 (1.1%)** | | |
| **Psychiatric disorders and or cognitive impairment** | **None** | | |
| **Preoperative anaemia** | **None** | | |
| **Preoperative interventions** |  | | |
| **A patient optimisation clinic** | **1 (0.5%)** | | |
| **Patient education** | **1 (0.5%)** | | |
| **Infection prevention** | **3 (1.6%)** | | |
| **Optimization of preoperative analgesia regimen** | **2 (1.1%)** | | |
| **Minimise preoperative fasting** | **4 (2.1%)** | | |
| **Establish a patient blood management programme** | **None** | | |
| **Intraoperative interventions** |  | | |
| **Meticulous surgical technique** |  | | |
| - **Operative time** | **4 (2.1%)** | | |
| **Infection prevention** |  | | |
| - **Antibiotics ≤ 30 min from skin cut** | **1 (0.5%)** | | |
| **Multimodal opioid-sparing analgesia regimen** |  | | |
| - **Peripheral nerve block by anaesthetist** | **None** | | |
| - **Local infiltration analgesia by surgeon** | **10 (18.6%)** | | |
| **Central neuraxial anaesthesia** | **None** | | |
| **Establish a patient blood management programme** |  | | |
| - **Tranexamic acid administration** | **1 (0.5%)** | | |
| - **Red blood cell transfusion** | **1 (0.5%)** | | |
| **Temperature regulation** | **2 (1.1%)** | | |
| **Postoperative interventions** | **Day 1** | **Day 2** | **Day 3** |
| **Early mobilisation after surgery** |  |  |  |
| - **Out of bed mobilisation** | **5 (2.7%)** | **11 (5.9%)** | **29 (15.6%)** |
| **Multimodal opioid-sparring analgesia regimen** | **5 (2.7%)** | **11 (5.9%)** | **28 (15.1%)** |
| **DVT prophylaxis** | **11 (5.9%)** | **11 (5.9%)** | **29 (15.6%)** |
| **A pain management team** | **7 (3.7%)** | **15 (8.1%)** | **15 (8.1%)** |
| **Patient controlled analgesia** | **5 (2.7%)** | **15 (8.1%)** | **15 (8.1%)** |
| **Physiotherapy** |  |  |  |
| **‘Timed up and go’ test** | **8 (4.3%)** |  |  |
| **Independent mobilization/readiness for discharge** | **13 (7.0%)** |  |  |
| **Orthopaedic discharge** |  |  |  |
| **In-hospital complications** | **None** |  |  |
| **Discharge destination** | **None** |  |  |
| **DAH_30_** |  |  |  |
| **Length of stay in hospital** | **None** |  |  |
| **Discharge destination** | **None** |  |  |
| **Readmission** | **None** |  |  |

| Data are n (%). |  |  |  |  |
| --- | --- | --- | --- | --- |

1. Myles, P.S., et al., *Validation of days at home as an outcome measure after surgery: a prospective cohort study in Australia.* BMJ Open, 2017. **7**(8): p. e015828.

2. Yeung, T.S., et al., *The timed up and go test for use on an inpatient orthopaedic rehabilitation ward.* J Orthop Sports Phys Ther, 2008. **38**(7): p. 410-7.

3. Jammer, I., et al., *Standards for definitions and use of outcome measures for clinical effectiveness research in perioperative medicine: European Perioperative Clinical Outcome (EPCO) definitions: a statement from the ESA-ESICM joint taskforce on perioperative outcome measures.* Eur J Anaesthesiol, 2015. **32**(2): p. 88-105.

4. Plenge, U., et al., *Optimising perioperative care for hip and knee arthroplasty in South Africa: a Delphi consensus study.* BMC Musculoskelet Disord, 2018. **19**(1): p. 140.
